# The draft genome of *Brucella abortus* strain Ba col-B012, isolated from a dairy farm in Nariño, Colombia, bring new insights into the epidemiology of biovar 4 strains

**DOI:** 10.1186/s40793-017-0299-2

**Published:** 2017-12-22

**Authors:** Mauricio Pacheco-Montealegre, Rocío E. Patiño, Ligia Torres, Sabrina Jiménez, José Luis Rodríguez, Alejandro Caro-Quintero

**Affiliations:** Corporación Colombiana de Investigación Agropecuaria - Corpoica. Centro de Investigación Tibaitatá, Mosquera - Bogotá, Cundinamarca Colombia

**Keywords:** *Brucellosis*, Abortion, Pathogen, Zoonosis

## Abstract

Brucellosis is a commonly diagnosed zoonosis that causes infertility and abortion in cattle, it is acquired from handling of infected animals or consuming contaminated milk or milk products. In Colombia, it belongs to the official notifiable disease list, despite its relevance little is known about the origin, epidemiology and the genetic constituents of the strains circulating in dairy farms. Here we present the draft genome of *B. abortus* Ba Col-B012, an isolate obtained from a female Holstein belonging to a dairy farm in Nariño, Colombia. This genome comprises 3,234,714 bp and 3018 predicted protein-encoding genes. Using comparative genomics and phylogenetic analysis, we found that the strain Ba Col-B012 clustered with known biovar 4 variants. The analysis of the core genes allowed the identification of polymorphisms only present in biovar 4 genomes, these regions are proposed as possible targets for identification by PCR. The sequencing of *B. abortus* Ba Col-B012 genome provides important insights to improve the diagnosis and the epidemiology of this disease and represents the first report of the biovar 4 in Colombia.

## Introduction

The brucellosis is one of the most important zoonotic diseases that causes infertility and abortion in cattle. In livestock, brucellosis is mainly caused by 10.1601/nm.1382, a Gram-negative coccobacillus that behaves as a facultative intracellular pathogen. There are up to eight variants of this species that differ on their physiological characteristics and are classified as biovars. However, some of these biovars differ only slightly and their status as true variants is unresolved. Some biovars have a wide geographic distribution; 10.1601/nm.1382 biovar1 and biovar2 are found around the world, while others as the biovar 5 are mainly distributed in Europe [1]. In South America, recent studies have identified several biovars, for instance, a survey of a 30-year 10.1601/nm.1382 collection from Brazil, found biovars 1, 2, and 3 [2], while in Ecuador, biovar 1 and 4 have been reported [3]. However, there still is a lack of sufficient information to establish biovar presence and distribution in other countries of the continent. In Colombia, even though there are regions with high prevalence and isolation of 10.1601/nm.1382 [4, 5], there are no reports on the identification of their corresponding biovars.

The genome presented here belongs to a larger collection of pathogens isolated as part of a monitoring program to identify the principal infectious agents related to infertility and abortion in cattle present in the southern part of Colombia [6]. During this survey, 12 10.1601/nm.1382 strains were isolated from dairy farms (Nariño, Colombia). Recently some of these strains were typified using AMOS-ERY-PCR [7] and MLVA methods [8], and a representative isolate was chosen for sequencing. Here we present the draft quality genome of the strain, 10.1601/nm.1382 Ba Col-B012, this genome contributes to a better understanding of the genomic constituents of local isolates and to the identification of virulence factors and conserved genes that code for immunogenic proteins that can eventually be used in the development of vaccines and new serological tests.

## Organism information

### Classification and features

**Fig. 1:**
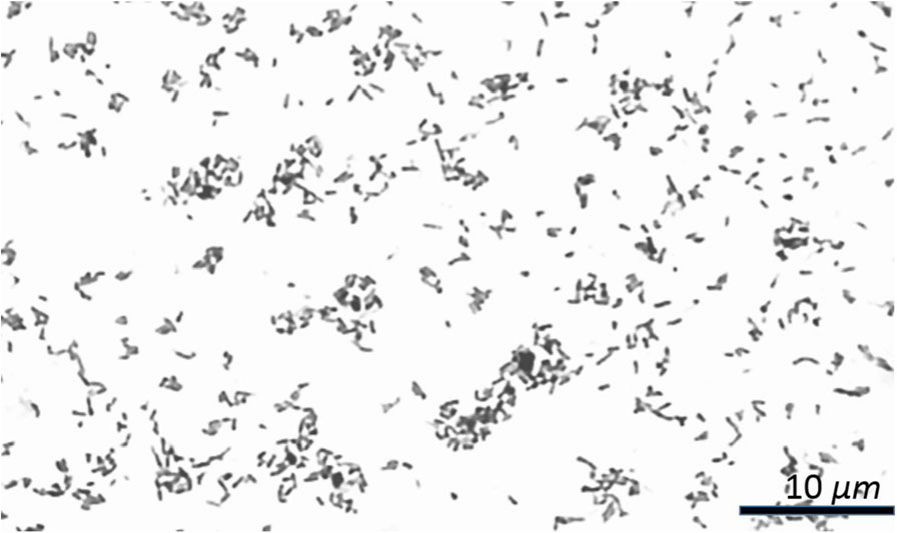
Photomicrograph of cells of *B. abortus* strain Ba Col-B012. Cells were grown on trypticase soy agar and brain infusion agar supplemented with 5% horse serum, this media was incubated at 37 °C for 48 h

10.1601/nm.1382 is a non-motil, Gram-negative short bacillus measuring about 0.6 to 1.5 μm by 0.5–0.7 μm (Fig. 1). The 10.1601/nm.1382 species belong to the family 10.1601/nm.1379, class 10.1601/nm.809 and phylum 10.1601/nm.808. Colonies are smooth, small, round, convex, and non-pigmented, on 10.1601/nm.1380 agar small colorless punctate colonies, appear within 48 to 72 h at 37 °C. Even though they are aerobes, providing a CO_2_ atmosphere may enhance growth.

The 10.1601/nm.1382 Ba Col-B012 strain was obtained from a female Holstein with an episode of abortion. The sample was taken from vaginal fluids with a swab and isolation was done on trypticase soy agar and brain infusion agar supplemented with 5% Horse serum, this media was incubated at 37 °C for 72 to 96 h, with a 5% CO_2_ atmosphere. Small transparent colonies were obtained with regular edges. Isolates were characterized by being non-motile and positive for the urease and oxidase tests and for the agglutination test by using polyclonal anti-10.1601/nm.1382 antibody (Difco). A summary of the classification and general features of 10.1601/nm.1382 strain Ba Col-B012 is presented in Table 1.

**Table 1:** Classification and general features of *B. abortus* strain Ba Col-B012

MIGS ID	Property	Term	Evidence code^a^
	Classification	Domain *Bacteria*	TAS [25]
		Phylum *Proteobacteria*	TAS [25, 26]
		Class *Proteobacteria alfa*	TAS [27]
		Order *“Rhizobiales”*	TAS [28]
		Family *Brucellaceae*	TAS [29, 30]
		Genus *Brucella*	TAS [30, 31]
		Species *Brucella abortus*	TAS [30, 31]
		strain: Col-B012	IDA
	Gram stain	Negative	TAS [31]
	Cell shape	Coccobacilli	TAS [31]
	Motility	Non-motile	TAS [31]
	Sporulation	Non-sporulating	TAS [31]
	Temperature range	20–40 °C	IDA
	Optimum temperature	37 °C	TAS [31]
	pH range; Optimum	6.6–7.4	TAS [31]
	Carbon source	d-glucose, d-ribose, l-malate, dl-lactate	TAS [32]
MIGS-6	Habitat	Holstein cattle	TAS [6]
MIGS-6.3	Salinity	–	NAS
MIGS-22	Oxygen requirement	Facultative	TAS [31]
MIGS-15	Biotic relationship	Host-associated	TAS [6]
MIGS-14	Pathogenicity	Pathogenic	NAS
MIGS-23	Isolation		IDA
MIGS-4	Geographic location	Nariño, Colombia	IDA
MIGS-5	Sample collection	June, 1997	IDA
MIGS-4.1	Latitude	00° 52’ N	IDA
MIGS-4.2	Longitude	−77° 39’ W	IDA
MIGS-4.4	Altitude	2900 m a.s.I	IDA

^a^Evidence codes - *IDA*: Inferred from Direct Assay; *TAS*: Traceable Author Statement (i.e., a direct report exists in the literature); *NAS*: Non-traceable Author Statement (i.e., not directly observed for the living, isolated sample, but based on a generally accepted property for the species, or anecdotal evidence). These evidence codes are from the Gene Ontology project [12/17]

## Genome sequencing and information

### Genome project history

10.1601/nm.1382 strain Ba Col-B012 was isolated as part of a monitoring program to identify the principal infectious agents related to infertility and abortion in cattle present in the southern part of Colombia [6]. The main objective for sequencing 10.1601/nm.1382 genomes is to explore the genomic constituents of the local isolates and to identify virulence factors, polymorphic regions, and immunogenic proteins that can be eventually be used in the development of vaccines and new serological and molecular tests. A summary of the project information is shown in Table 2.

**Table 2:** Project information

MIGS ID	Property	Term
MIGS 31	Finishing quality	Improved high-quality draft
MIGS-28	Libraries used	One Illumina paired-end
MIGS 29	Sequencing platforms	Illumina HiScan SQ
MIGS 31.2	Fold coverage	50 × Illumina
MIGS 30	Assemblers	Newbler 2.0.01.14
MIGS 32	Gene calling method	GeneMarkS+, Glimmer, Prodigal
	Locus Tag	LODQ01
	Genbank ID	LODQ01000000.
	GenBank Date of Release	01/09/2017
	GOLD ID	_
	BIOPROJECT	PRJNA305302
	Project relevance	Host-associated

### Growth conditions and genomic DNA preparation

10.1601/nm.1382 strain Ba Col-B012 strain was grown on trypticase soy agar and brain infusion agar supplemented with 5% horse serum, this media was incubated at 37 °C for 72 h. Genomic DNA extraction was done with the CTBA-Phenol Chloroform method couple to ethanol precipitation [9]. DNA was quantified using the dsDNA HS (High Sensitivity) kit on a Qubit™ (Life Technologies), a greater than 30 ng/μl DNA concentration was obtained. Quality and purity of DNA was determined by spectrophotometry (Nanodrop® 2000 Thermo Fisher Scientific) obtaining a 260/280 and 260/230 ratio equal to 2.

### Genome sequencing and assembly

Whole-genome sequencing of the 10.1601/nm.1382 strain Ba Col-B012 strain was performed by employing the Illumina HiScan SQ (Molecular Biology Lab, Corpoica). Libraries were generated using the Sure Select Strand Agilent Sample Preparation, once the DNA concentration was determined library amplification was done with the TruSeq PE Cluster Kit v3, (Illumina), using Cbot (Illumina). For de novo assembly, we used 3,956,238 paired-end Illumina reads (150 bp) and the Newbler v 2.0.01.14 software. The assembly resulted in 233 contigs with total genome length of 3227,565 bp and with 50× average coverage.

### Genome annotation

Gene prediction was conducted with GeneMarkS+ [10], and PRODIGAL [11] and annotation was done automatically using the NCBI Prokaryotic Genome Annotation Pipeline. The annotation was corrected manually using the data from different databases (Swiss-Prot [12] and RAST [13]). We use LipoP v 1.0 [14] for finding genes with signal peptides and with transmembrane helices.

## Genome properties

The genome statistics are provided in Table 3. The assembly resulted in 233 contigs with total genome length of 3227,565 bp and with 50× average coverage. The N_50_ contig size is 22,624 and a maximum contig size of 106,301 bp and a G + C content of 57.28 mol%. These values are similar to those reported for the genomes NC_006932.1, NZ_CP007709.1 and NZ_CP007705.1 of 10.1601/nm.1382 at NCBI. Using our annotation pipeline, it was possible to identify 3227 predicted genes of which 3018 were putatively protein-encoding, 166 pseudogenes, 42 tRNAs and 1 ncRNA. For the majority of the protein-encoding genes (78.12%) a function could be assigned. The distribution of these genes into COG functional categories [15] is shown in Table 4. This Whole Genome Shotgun project has been deposited at DDBJ/ENA/GenBank under the accession LODQ00000000. The version described in this paper is version LODQ01000000.

**Table 3:** Genome statistics of *B. abortus* strain Ba Col-B012

Attribute	Value	% of Total
Genome size (bp)	3,234,714	100.00
DNA coding (bp)	2,685,762	83.02
DNA G + C (bp)	1,472,070	45.50
DNA scaffolds	243	100.00
Total genes	3227	100.00
Protein coding genes	3018	93.52
RNA genes	42	1.30
Pseudo genes	166	5.14
Genes in internal clusters	164	5.43
Genes with function prediction	2408	74.62
Genes assigned to COGs	2521	78.12
Genes with Pfam domains	2631	81.53
Genes with signal peptides	380	11.77
Genes with transmembrane helices	422	13.07
CRISPR repeats	0	0

**Table 4:** Number of genes associated with general COG functional categories

Code	Value	%age	Description
J	160	5.30	Translation, ribosomal structure and biogenesis
A	0	0	RNA processing and modification
K	193	6.39	Transcription
L	117	3.87	Replication, recombination and repair
B	1	0.03	Chromatin structure and dynamics
D	28	0.92	Cell cycle control, Cell division, chromosome partitioning
V	50	1.65	Defense mechanisms
T	79	2.61	Signal transduction mechanisms
M	137	4.53	Cell wall/membrane biogenesis
N	30	0.99	Cell motility
U	23	0.76	Intracellular trafficking and secretion
O	98	3.24	Posttranslational modification, protein turnover, chaperones
C	169	5.59	Energy production and conversion
G	177	5.86	Carbohydrate transport and metabolism
E	307	10.17	Amino acid transport and metabolism
F	73	2.41	Nucleotide transport and metabolism
H	107	3.54	Coenzyme transport and metabolism
I	93	3.08	Lipid transport and metabolism
P	200	6.62	Inorganic ion transport and metabolism
Q	36	1.19	Secondary metabolites biosynthesis, transport and catabolism
R	0	0	General function prediction only
S	481	15.93	Function unknown
–	497	16.46	Not in COGs

The total is based on the total number of protein coding genes (3018) in the genome

## Insights from genome sequences

### Genomes used in this study

A total of 28 10.1601/nm.1382 genomes were downloaded from the NCBI database of complete and draft bacterial genomes, even though there are many more genomes in the database, only those with identified biovar were used for further analyses. The genomes and their GeneBank accession numbers are listed in Table 5. The genes used in the analysis were predicted from the genomes using PRODIGAL with the default settings [11].

**Table 5:** Genomes and accession numbers used in this study

Biovar	Strain name	Genome assembly number
1	*Brucella abortus* biovar 1 NI435a	GCA_000245835.1
1	*Brucella abortus* biovar 1 NI486	GCA_000245855.1
1	*Brucella abortus* biovar 1 NI474	GCA_000245875.1
1	*Brucella abortus* biovar 1 NI488	GCA_000245895.1
1	*Brucella abortus* biovar 1 NI010	GCA_000245915.1
1	*Brucella abortus* biovar 1 NI016	GCA_000245935.1
1	*Brucella abortus* biovar 1 NI021	GCA_000245955.1
1	*Brucella abortus* biovar 1 NI259	GCA_000245975.1
1	*Brucella abortus* biovar 1 str 134	GCA_000298635.1
1	*Brucella abortus* biovar 1 76–1413	GCA_000413495.1
1	*Brucella abortus* biovar 1 84–0928	GCA_000413575.1
1	*Brucella abortus* biovar 1 90–0742	GCA_000413655.1
1	*Brucella abortus* biovar 1 94–1313	GCA_000413735.1
1	*Brucella abortus* biovar 1 01–0648	GCA_000413755.1
1	*Brucella abortus* biovar 1 01–0585	GCA_000413775.1
1	*Brucella abortus* biovar 1 01–0065	GCA_000413795.1
1	*Brucella abortus* biovar 1 B10–0018	GCA_000413815.1
1	*Brucella abortus* biovar 1 B10–0091	GCA_000413955.1
1	*Brucella abortus* biovar 1 89–0363	GCA_000413975.1
1	*Brucella abortus* biovar 1 87–2211	GCA_000413995.1
1	*Brucella abortus* biovar 1 82–2330	GCA_000414015.1
1	*Brucella abortus* biovar 1 80–1399	GCA_000478665.1
2	*Brucella abortus* biovar 2 82–3893	GCA_000413555.1
2	*Brucella abortus* biovar 2 90–0737	GCA_000413695.1
2	*Brucella abortus* biovar 2 90–1280	GCA_000413715.1
4	*Brucella abortus* biovar 4 68-3396P	GCA_000413535.1
4	*Brucella abortus* biovar 4 90–0775	GCA_000413675.1
4	*Brucella abortus* biovar 4292	GCA_000157695.1

### Genomic differences between 10.1601/nm.1382 BA col-B012 and the type strain 10.1601/nm.1382 2308

The comparative genomic analysis between 10.1601/nm.1382 strain BA Col-B012 and the type strain, 10.1601/nm.1382 2308, shows that both genomes shared 3015 genes, most of these genes are identical (2862 genes with 100%). Within this set of genes there are around 12 genes that are divergent with a nucleotide identity ranging from 77 to 94%, (Additional file 1: Table S1) among the genes are an ABC transporter permease, benzoate transporter, an alpha/beta hydrolase, a 5-hydroxymethyluracil DNA glycosylase, several hypothetical proteins and a hemolysin D gene (HlyD). Hemolysin D is part of the membrane transporter of the HlyA, a pore-forming toxin that affects the membrane of the host [16]. We also identified 16 genes present in strain BA Col-B012 that were not found in the type strain (Additional file 1: Table S2). Most genes in this set are hypothetical proteins, transporters and transcriptional regulators. These differences show that strain BA Col-B012 differs from the type strain 2308. In order to elucidate if these differences are related to the biovar classification a comparative genomic analysis with more strain was done in the next section.

### The evolutionary distance and phylogenetic relationship of 10.1601/nm.1382 strain Ba col-B012

A phylogenomic approach was done to establish the evolutionary relationship of 10.1601/nm.1382 strain Ba Col-B012 and to evaluate whether biovars are congruent with true genetic groupings. The phylogenetic analysis was done by concatenating the alignment of orthologues genes shared by all strains. In order to identify a set of orthologous genes, an in-house PERL script that incorporates the reciprocal best match approach was used [17]. In brief, the predicted genes of strain Ba Col-B012 were searched using the blastn algorithm [18] against the genomic sequences of each of the remaining genomes. The best match for each query gene (genes with higher than 70% identity and alignment coverage) was extracted and searched against the complete gene complement of the Ba Col-B012 strain to identify reciprocal best matches. The reciprocal best match genes were denoted as orthologues, 3139 orthologous genes were shared among all strains, from these 2169 were identical among all strains (100% nucleotide identity). Average nucleotide identity (ANI) was quantified using the nucleotide identity of orthologues between the strain Ba Col-B012 and the other genomes, this is a measurement of genomic divergence that is used in modern taxonomy as the gold standard to delimitate new species [19, 20]. The ANI values between the Ba Col-B012 and the rest of the strains were higher than 99.6 % (Additional file 1), these high identity reflect the close evolutionary relationship between the 10.1601/nm.1382 strains that make difficult the identification of biovar variants. Despite the close relationship between all genomes, strain Ba Col-B012 showed a closest affiliation with biovar 4 strains (99.88%).

**Fig. 2:**
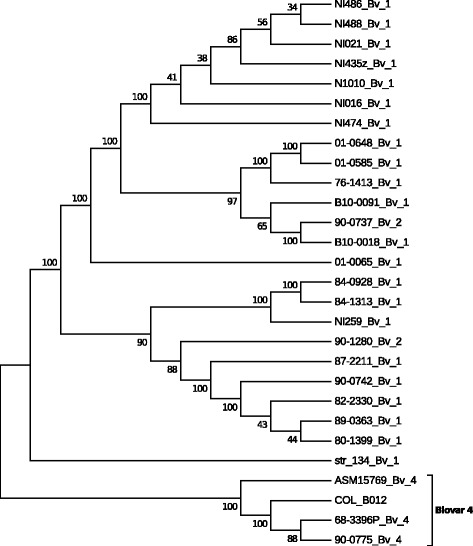
Evolutionary relationships of *B. abortus* strain Ba Col-B012. The evolutionary history was inferred using the Neighbor-Joining method [33]. The bootstrap consensus tree inferred from 1000 replicates [34] is taken to represent the evolutionary history of the taxa analyzed [35]. All positions containing gaps and missing data were eliminated. There were a total of 2,632,124 positions in the final dataset. Evolutionary analyses were conducted in MEGA6 [36]

In order to corroborate the affiliation of Ba Col-B012 to biovar 4, the phylogenetic relationship of shared polymorphic genes, around 2961 genes, was inferred using the Neighbor Joining algorithm with the Jukes-Cantor distance and 1000 bootstraps (Fig. 2). As shown before by the ANI analyses, strain Ba Col-B012 was more closely related to the biovar 4 strains clustering in the same clade with a 100 bootstrap value. This represents the first confirmed report of a biovar 4 strain in Colombia, and may suggest a possible transfer from Ecuador which is the country that delimits with the Nariño region and where biovar 4 has been reported [3].

### Used of polymorphic regions in the identification of 10.1601/nm.1382 Biovar 4 and its potential for diagnosis and vaccination

Current identification of biovars is based on standard microbiological methods and molecular approaches like MLVA analysis. MLVA is particularly a high discriminatory method useful in epidemiological studies and in the identification of genetic variability of strains [21]. However, this methodology is not always conclusive. In order to complement the current methods of diagnosis with PCR-based amplification and sequencing, orthologous regions that could be used to differentiate biovar 4 genomes from others were identified. We found around 42 genes with polymorphism that differentiate biovar 4 genomes from the rest. Most genes have only one single nucleotide polymorphism (SNP), from this set almost half of the SNPs are non-synonymous. From all evaluated genes, only one hypothetical gene has two polymorphisms that are synonymous (set 12). We also found two genes that have insertion-deletions and three genes that are shorter than the biovar 1 counterpart due to the presence of an early stop codon (See Table 6 for a description of genes and differences). All gene set described in the analysis are provided in the Additional files section.

**Table 6:** Analysis of polymorphic genes that differentiate biovar 4 from other genomes

Set	Annotation	Type	Description
0	The major facilitator superfamily (MFS) is a class of membrane transport proteins	Syn	T-G (pos 87)|
1	Hypothetical protein	Syn	T-G (pos 283)
2	Multiple antibiotic resistance transporter	NonSyn	C-T (pos 424), P-S (pos 142)
3	Calcium/calmodulin dependent protein kinase II	Syn	C-A (pos 95)
4	Peptidase Do	Syn	C-A(pos 829)
5	30S ribosomal protein S14	Syn	C-A(pos 124)
6	Hypothetical protein	NonSyn	G-T (pos 186), Q-H (pos 62)
7	Excinuclease ABC subunit B	Stop	STOP codon
8	Hypothetical protein similar with BA14K family domain	In/Del	IN/DEL 12 nuc (pos 327)
9	Flagellar basal body rod protein FlgC	NonSyn	G-A (pos 55), A-T (pos 19)
10	Dipeptide ABC transporter permease DppC	NonSyn	T-C (pos 478), S-P (pos 160)
11	Na(+)/H(+) antiporter NhaA	Stop	IN/DEL-ORF G?- (pos 1917)
12	Hypothetical protein	Syn	G-A (pos 609), C-T (pos 633)
13	DNA-3-methyladenine glycosylase	Syn	C-T (pos 483)
14	Mannosyltransferase	NonSyn	A-C(pos 980), K-N (pos 349)
15	Hypothetical protein	NonSyn	C-T(pos 229), T-I (pos75)
16	Class II fumarate hydratase	NonSyn	C-T(pos 1323), A-V (pos 441)
17	Hypothetical protein	Syn	G-C(pos 250)
18	Acyl carrier protein	Syn	C-T (pos 260)
19	Tyrosine--tRNA ligase	Syn	C-G(pos1107)
20	Glycosyl transferase	NonSyn	C-T(pos 35), V-A(pos12)
21	D-alanyl-D-alanine carboxypeptidase	NonSyn	A-G(pos 451), T-A (pos 151)
22	Malic enzyme	Syn	T-C (pos723)
23	X-Pro dipeptidase		T-C(pos 280), F-L (pos 94)
24	Putative multidrug efflux transporter protein	Stop	G-T(pos 229), E-STOP
25	D-ribose ABC transporter substrate-binding protein	NonSyn	C-T(pos 396), A-V(pos 132)
26	NAD-dependent dehydratase	NonSyn	A-G(pos196), M-V (pos 66)
27	Phosphogluconate dehydratase	NonSyn	C-T(pos620), A-V(pos 207)
28	Hypothetical protein	Syn	C-T (pos628)
29	Hypothetical protein	NonSyn	A-G(pos235), T-A (pos 79)
30	Glutamine synthetase	Syn	G-A(pos 1306)
31	N-formylglutamate amidohydrolase	Syn	A-G (pos 541)
32	Hypothetical protein	NonSyn	A-T(pos 36), K-M(pos12)
33	Branched-chain amino acid ABC transporter, ATP-binding/permease protein	NonSyn	A-G(pos452), N-S(pos 151)
34	DNA topoisomerase	NonSyn	C-A(pos 1827),
			R-S(pos 609)
35	Aspartate carbamoyltransferase	Syn	A-G(pos 540)
36	8-amino-7-oxononanoate synthase	NonSyn	A-G(pos 991), R-G(pos 331)
37	Secretion protein HlyD family protein-hemolysin secretion protein D	NonSyn	G-A(pos 415), V-I(pos 139)
38	Tetracycline resistance protein TetB	NonSyn	T-G(pos 765), F-L(pos 225)
39	Mannose-1-phosphate guanylyltransferase/mannose-6-phosphate isomerase	NonSyn	G-T(pos 590), F-C(pos 197)
40	ABC transporter permease	In/Del	Large Insertion of up to 43 aa
41	Aminobutyraldehyde dehydrogenase	Syn	T-C (pos 342)

Position are relative to the gene set alignment. Alignments are provided as Additional file 2

In order to design primers for genetic markers for biovar 4, we focused on orthologues amplifiable by PCR (<400 bp) that have large INDELs or genes with synonymous polymorphisms, this guarantees that the observed changes are not under selection. We identified six genes that met this criteria, these were: hypothetical protein similar with BA14K family domain (gene set 8), hypothetical protein (gene set 12), 10.1601/strainfinder?urlappend=%3Fid%3DDNA+3-methyladenine glycosylase (gene set 13), tyrosine--tRNA ligase (gene set 19), glutamine synthetase (gene set 30), and ABC transporter permease (gene set 40). Based on these genes, we designed sets of primers that amplify the polymorphic regions and therefore can be used for the identification. Table 7 summarizes the designed primers and their predicted PCR conditions.

**Table 7:** Designed primer sets to differentiate biovar 4 from others

Set	F. pos (bp)	R. pos (bp)	Forward primer	Reverse primer	Tm (°C)
8	281	435	AGCCACGCACGACCTATATC	GCCCGAGCAATACTGATACC	60
12	478	877	GAAGCCGATCAGCAATTCAC	AAAGCAGGATCGCCACATAG	60
13	178	552	GGATTGTCGTGGCTTACGAT	GAAGGCATAGACCGTGGTTG	60
19	962	1218	ACGCAAGACCTTTGAAGACG	GAGCGACAGCTTGATGAGG	60
30	923	1322	CGCCTTACATCAATTCCTACAA	CGGTCATATTCGATCTGTTCC	59
40	22	598	ATTCTCGATCCGCATTTCAT	AGAGGCCGGAGAGAATAAGC	60

Position of primers is relative to the gene set alignment

Comparative genome analysis of 10.1601/nm.1382 strains is a powerful tool for the identification of allele variants/polymorphism that modulate virulence. Interestingly, among the identified polymorphic genes, two genes have been associated with pathogenicity and immune response, a hypothetical protein similar with BA14K family domain (Table 6, gene set 8) and a gene coding for the subunit B of the exonuclease ABC (Table 6, gene set 7). The domain 10.1601/strainfinder?urlappend=%3Fid%3DBAL+14K had been demonstrated to induce a strong immunoreactivity in mice, with a Th1 response and induction of IL-12 secretion [22]. While changes in the subunit B of exonuclease ABC have been associated with minor virulence changes between attenuated and virulent 10.1601/nm.1380 strains [23]. It is also worth mentioning that several other sets of genes identified as polymorphic might also display immunogenic reactivity, as their coding proteins are located in the membrane at the interphase with the environment, for instance, several transporters in 10.1601/nm.1382 have been used to produce in vivo-induced antigens [24]. These genes are potential targets for future vaccination and diagnosis.

## Conclusions

The genome of 10.1601/nm.1382 Ba Col-B012 contributes to the better understanding of the distribution and origin of zoonotic pathogens in Colombia and South America. A better representation of biovar genomes can be used to elucidate the correspondence between evolutionary relationship and phenotypic characteristics. The phylogenomic relationship between strain Ba Col-B012 and the examined genomes shows that biovar 4 strains form a distinctive clade with high bootstrap support. This pattern is not observed for other biovars, for example, strain 90–0737 and strain B10–0018, which cluster in the same clade, are classified into different biovar groups. The clear clustering of biovar 4 genomes reflects a common ancestor of the group and suggests the existence of allele differences that might be associated with the phenotypic and pathogenic characteristics of the group. Finally, the identification of biovar 4 distinctive genomic region allowed us to design sets of primers that coupled with sequencing could be incorporated into current methods of identification to distinguish biovar 4 strains from others. The 10.1601/nm.1382 Ba Col-B012 genome provides important insights to improve the diagnosis and the epidemiology of this disease and represents the first report of the biovar 4 in Colombia.

## Additional files

Additional file 1: Genes that differentiate *Brucella abortus* strain Ba Col-B012 from the type strain. (DOCX 56 kb)

Additional file 2: Alignment of polymorphic genes, sets 0–41. (ZIP 58 kb)

## Abbreviations

AAI: Average Amino Acid Identity
MLVA: Multiple-Locus Variable number tandem repeat Analysis.
