# Supplementary material for: The draft genome of Brucella abortus strain Ba col-B012, isolated from a dairy farm in Nariño, Colombia, bring new insights into the epidemiology of biovar 4 strains
Source: Stand Genomic Sci. 2017 Dec 22;12:89. doi: 10.1186/s40793-017-0299-2 (PMC5741917; doi:10.1186/s40793-017-0299-2)
Supplement: Supplementary file 1 — Genes that differentiate Brucella abortus strain Ba Col-B012 from the type strain. (DOCX 56 kb) [file 40793_2017_299_MOESM1_ESM.docx]

**Additional file 1**

**Figure S1. Average nucleotide identity distribution of strain Ba Col-B012 compared to the three different biovars.**

**
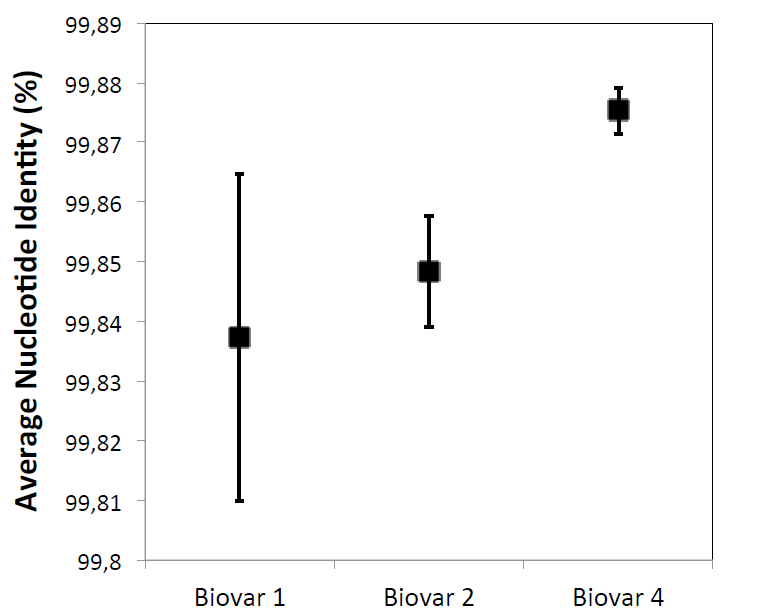
**

**Table S1. Divergent shared genes with the type strain**

| **Protein annotation** | **Protein id** | **Identity (%)** | |
| --- | --- | --- | --- |
| amino acid ABC transporter permease | OLS11662.1 | 76 |  |
| alpha/beta hydrolase | OLS07359.1 | 76 |  |
| ABC transporter permease | OLS07112.1 | 77 |  |
| LysR family transcriptional regulator] | OLS10285.1 | 85 |  |
| benzoate transporter | OLS07398.1 | 87 |  |
| hypothetical protein | OLS11809.1 | 89 |  |
| hypothetical protein | OLS08315.1 | 90 |  |
| hypothetical protein | OLS05400.1 | 93 |  |
| hypothetical protein | OLS05918.1 | 94 |  |
| ATPase | OLS10512.1 | 95 |  |
| hemolysin D | OLS11163.1 | 97 |  |
| 5-hydroxymethyluracil DNA glycosylase | OLS08807.1 | 98 |  |

**Table S2. Orthologous genes present in ColB012 and absence in the type strain**

| **Protein annotation** | **Protein id** |
| --- | --- |
| ABC transporter substrate-binding protein | OLS10744.1 |
| acetoacetyl-CoA synthetase | OLS05960.1 |
| acyltransferase | OLS05962.1 |
| CHRD domain-containing protein | OLS05961.1 |
| hypothetical protein | OLS08990.1 |
| hypothetical protein | OLS08336.1 |
| hypothetical protein | OLS06546.1 |
| hypothetical protein | OLS05967.1 |
| hypothetical protein | OLS05619.1 |
| hypothetical protein | OLS05092.1 |
| isovaleryl-CoA dehydrogenase | OLS05959.1 |
| MFS transporter | OLS10747.1 |
| NUDIX hydrolase | OLS06875.1 |
| transcriptional regulator | OLS10745.1 |
| transcriptional regulator | OLS10746.1 |
| transposase | OLS07681.1 |
